# Taming Cell-to-Cell Heterogeneity in Acute Myeloid Leukaemia With Machine Learning

**DOI:** 10.3389/fonc.2021.666829

**Published:** 2021-04-29

**Authors:** Yara E. Sánchez-Corrales, Ruben V. C. Pohle, Sergi Castellano, Alice Giustacchini

**Affiliations:** ^1^ Genetics and Genomic Medicine Department, Great Ormond Street Institute of Child Health, University College London, London, United Kingdom; ^2^ Molecular and Cellular Immunology Section, Great Ormond Street Institute of Child Health, University College London, London, United Kingdom; ^3^ University College London (UCL) Genomics, Great Ormond Street Institute of Child Health, University College London, London, United Kingdom

**Keywords:** AML, machine learning, classification, clustering, leukaemia

## Abstract

Acute Myeloid Leukaemia (AML) is a phenotypically and genetically heterogenous blood cancer characterised by very poor prognosis, with disease relapse being the primary cause of treatment failure. AML heterogeneity arise from different genetic and non-genetic sources, including its proposed hierarchical structure, with leukemic stem cells (LSCs) and progenitors giving origin to a variety of more mature leukemic subsets. Recent advances in single-cell molecular and phenotypic profiling have highlighted the intra and inter-patient heterogeneous nature of AML, which has so far limited the success of cell-based immunotherapy approaches against single targets. Machine Learning (ML) can be uniquely used to find non-trivial patterns from high-dimensional datasets and identify rare sub-populations. Here we review some recent ML tools that applied to single-cell data could help disentangle cell heterogeneity in AML by identifying distinct core molecular signatures of leukemic cell subsets. We discuss the advantages and limitations of unsupervised and supervised ML approaches to cluster and classify cell populations in AML, for the identification of biomarkers and the design of personalised therapies.

## Introduction

AML is an aggressive and fast-progressing leukaemia characterised by the accumulation of myeloid progenitors (1). Although most patients achieve remission after first line chemotherapy and haematopoietic stem cell transplantation, about 40% later relapse (2). Long-term survival following relapse is below 20% with a median survival of 4-6 months, an outcome that has not improved over the last two decades with conventional approaches (2–4) and novel therapies are therefore urgently needed (4).

**Figure 1:**
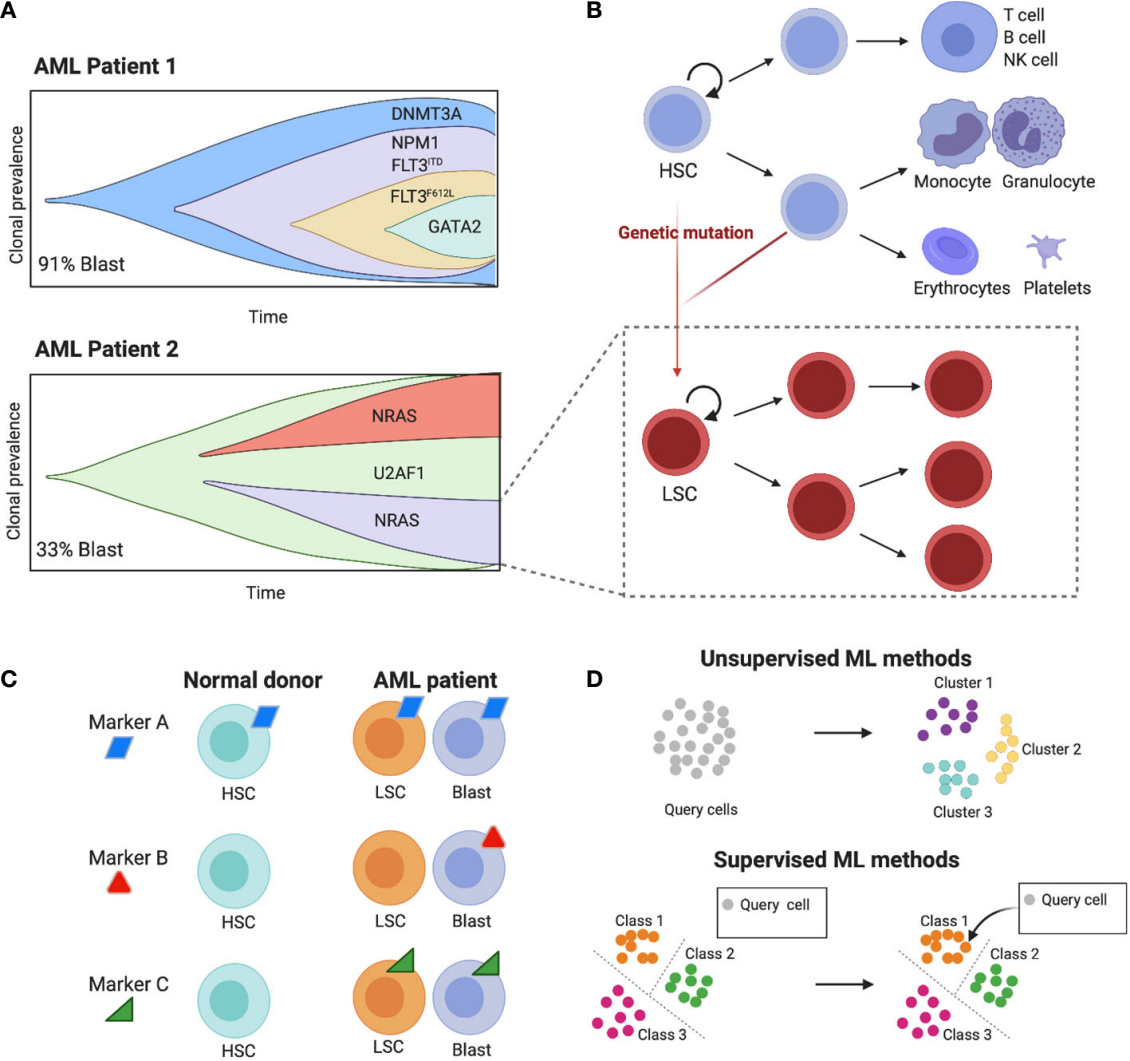
The high cell-to-cell heterogeneity in AML tumours can be dissected using machine learning methods. **(A)** The schematic representing clonal diversity in two putative AML patients highlights the complex intra and inter-patient variation of cell diversity (schematics adapted from Petti et al., 2019). Importantly, each clone carries its own hierarchical structure (here shown for one clone as an example). **(B)** Leukemic populations share the hierarchical organization of normal hematopoietic development, where hematopoietic stem cells (HSCs) differentiate into multiple cell lineages, giving rise to all mature blood cells (blue lineages). Genetic mutations induce malignant transformation and give rise to leukemic stem cells (LSCs) that share some characteristics of their normal counterparts such as unlimited ability to self-renew and the potential to give origin to a variety of more mature leukemic subsets (red lineages). **(C)** Ideal targets for immunotherapy with engineered T cells are those present in both leukemic blast and LSC cells and absent in healthy cell types. Targets that are ubiquitously expressed will fail to target specific leukemic populations and will be toxic for normal cells (on target off, tumour toxicity). Targets that are absent from LSC will render the treatment prone to relapse. Due to the high cell heterogeneity in AML more than one molecule is likely to fulfil these requirements. **(D)** Machine learning methods to identify cell populations can be unsupervised and supervised. The former uses the intrinsic structure of the data to cluster cells in an automatic fashion. The second uses a predefined set of groups to classify unknown cells, leveraging previous knowledge. Figure created with BioRender.com.

AML is a molecularly heterogeneous group of diseases with a complex mutational landscape, characterised by intra- and inter-patient variation (**Figure 1A**). Advances in next-generation sequencing and single-cell technologies have revealed that AML cells display genetic and epigenetic heterogeneity in different patients and even within the same patient multiple sub-clones co-exist, each carrying its own hierarchical structure and possessing distinct immunophenotypes (5).

A non-genetic source of heterogeneity in AML is its proposed hierarchical structure, mimicking the cellular hierarchy in normal hematopoietic development (**Figure 1B**). In healthy individuals, this involves a stepwise differentiation process, with hematopoietic stem cells (HSCs) giving rise to progressively more mature blood cells (6–8). LSCs lie at the top of AML cellular hierarchies, and carry an unlimited ability to self-renew as well as giving origin to a variety of more mature leukemic subsets (1), each expressing characteristic patterns of cell surface markers. LSCs can persist in a dormant state, making them selectively unresponsive to conventional chemotherapies and allowing them to eventually fuel disease relapse. For these reasons, the effective targeting of LSCs underpins any successful treatment for AML.

A promising approach is to target LSCs using immunotherapy with autologous T cells genetically redirected to express Chimeric Antigen Receptors (CARs). In fact, CAR-T cells can effectively target tumour cells irrespectively of their quiescent status. However, the lack of surface markers preferentially expressed on LSCs as opposed to healthy HSCs has hindered the development of cell-based immunotherapy strategies for AML, given the high risk of on-target off-tumour toxicity (9, 10). In addition, some of the targets tested so far (e.g. CD33 or CD123) have heterogenous expression in the LSC compartment, with the risk of relapse due to their incomplete targeting (11). Upon relapse, genetic and immunophenotypic heterogeneity in AML LSCs further increases, complicating the discovery of ‘one fits all’ drug target (12).

As a result of AML’s heterogenous nature, CAR-T cell approaches against a single target are unlikely to be effective, thus the design of combinations of CAR-T cells against multiple targets requires a systematic characterization of the expression levels of surface antigens in AML cell populations at single-cell resolution (**Figure 1C**) (9).

The unprecedented resolution achieved with single-cell technologies has enabled the dissection of cell populations, including tumour and rare cell types that could not be identified using conventional bulk sequencing (13, 14). In AML, the quantitative phenotyping of leukemic cell profiles has allowed the identification of leukemic subsets without prior knowledge of phenotypic markers for their prospective isolation, opening up new analytical challenges for their clinical interpretation (5, 15–19).

Despite Machine Learning (ML) techniques having shown prognostic utility in classifying patients at high risk of relapse and having been applied to risk-adapted treatments [review by (20)], they have only been recently applied to resolve heterogeneity in single-cell datasets from AML patients (15, 18). Fortunately, there has been an explosion of new algorithms based on ML for the characterization of cell populations in single-cell datasets (**Table 1**) that could be applied to identify molecular markers specific to AML subpopulations.

**Table 1:** Summary of recent ML-based methods to identify cell types.

Algorithm name	Classification type	Method	Input data	Important contribution	Reference
SC3	Unsupervised	Consensus clustering and hierarchical clustering	Normalised expression matrix	Transcriptome-based identification of genetic subclones in myeloproliferative neoplasms	(21)
cNMF	Unsupervised	Non-negative matrix factorization	Expression matrix and several parameters	Identification of previously misclassified immature skeletal muscle cells in a published dataset from brain organoids	(22)
scCOGAPS	Unsupervised	Non-negative matrix factorization	Normalised and log-scaled expression matrix	Identification of gene expression signatures characteristic of discrete cell types in the developing retina	(23)
SCCAF	Unsupervised	Logistic Regression and self-projection	Expression matrix and several parameters	Identification of cell states associated with different stages of erythroid maturation in mouse	(24)
WNN	Unsupervised	K-nearest neighbours and Jaccard distance	Expression matrix and protein matrix (or any other single-cell measurement)	Single-cell multimodal analysis improves resolution of cell states in the immune system and identify previously unreported subpopulations	(25)
CellAssign	Supervised	Expectation-Maximization hierarchical model	List of cell markers, subset of expression matrix containing the marker genes and some parameters	Resolution of malignant and non-malignant cells and their molecular dynamics during disease progression in follicular lymphoma	(26)
Garnett	Supervised	Multinomial elastic-net regression	Hierarchical list of cell markers (positive and negative) and expression matrix	The model trained on a mouse lung dataset is successfully applied to detect both healthy cell types and tumor cells in a human lung cancer dataset	(27)
scmap	Supervised	k-means (scmap-cluster) and k-nearest-neighbour (scmap-cell)	Annotated reference dataset and query expression matrix	Cell types in a test datasets are annotated with high accuracy irrespectively of batch effect	(28)
CHETAH	Supervised	Hierarchical Spearman correlation	Annotated reference dataset and query expression matrix (both normalised and log –scaled)	The cell type identification algorithm correctly identifies cancer cells absent in the reference dataset as “unassigned” or “intermediate”	(29)
scClassify	Supervised	Hierarchical ordered partitioning, ensemble learning and weighted k-nearest-neighbour	Annotated reference dataset and query expression matrix (both log –transformed)	Identification of cell types from the Tabula Muris single cell dataset that were unidentified in the original publication, including very rare populations	(30)
SingleR	Supervised	Correlation to training set	Annotated reference dataset and query expression matrix (both normalised and log-transformed)	Identification of a subgroup of macrophages whose molecular markers are upregulated in samples from patients with idiopathic pulmonary fibrosis.	(31)
SingleCellNet	Supervised	Random Forest	Annotated reference dataset and expression matrix (both raw)	Cells from pancreatic tissue that were “unclassified” in the original study are identified as Schwann cells and gamma cells	(32)
SuperCT	Supervised	Artificial Neural Network	Pre-trained ANN model and a query expression matrix	The model predicts cell types with high accuracy in multiple single cell test datasets including cord blood mononuclear cells and mouse pancreatic cancer.	(33)
ACTINN	Supervised	Artificial Neural Network	Annotated reference dataset and query expression matrix	Model trained on a T cell subtype reference accurately predicts T cell subtypes from an independent peripheral blood mononuclear cells dataset	(34)
Moana	Supervised	Support Vector Machine	Pre-trained model and raw query expression matrix	Identification of common and cell type-specific gene expression responses to IFN-β treatment in peripheral blood cells	(35)
scPred	Supervised	Support Vector Machine	Annotated reference dataset and query expression matrix (both normalised)	Prediction of pathological cell states in gastric and colorectal cancer	(36)

Here, we review some recent state-of-the-art ML methods with the potential to shed light into cell heterogeneity in AML and identify biomarkers for specific cell populations in single-cell datasets. Benchmarking of some recent methods has been done by (37) and (38). Rather than an extensive discussion of algorithms, we provide a general overview of tools available to identify cell populations in single-cell studies, highlighting ones that have the potential to reveal new and rare cell types in AML and aid the design of personalised treatments.

## Machine Learning for Cell Type Identification in Single-Cell Datasets and Biomarker Discovery for Personalised Immunotherapy

Single-cell high-throughput techniques, such as scRNA-seq, quantitatively characterise **cell types** within a tissue (39). Typical workflows in single-cell transcriptional profiling include dimensionality reduction and clustering of cells based on their gene expression patterns followed by manual annotation of cell clusters from known cell type **markers** (40). In the context of AML and other cancers, transcriptionally similar malignant cells are expected to group together, and can be unambiguously identified by the expression of certain feature genes that can be used as biomarkers for designing personalised treatments.

The identification of cell types using typical workflows has several drawbacks: first, rare cell types are easily missed and grouped together with some more prevalent ones; second, cell identity is often not discrete but lies in a continuum (for instance, cells with mixed identities or in transition); and third, the clustering can reflect other sources of variability unrelated to cell types (41). To address these issues, ML tools have recently been developed allowing quantitative identification and probabilistic assignment of cell types, thus aiding the identification of rare and heterogeneous cell populations.

In general, ML approaches are either **unsupervised** or **supervised** (**Figure 1D**). The main difference being the use of prior knowledge. Supervised methods are **trained** on an **annotated reference** with known **classes** of cell types, whereas unsupervised models identify patterns in the data without prior knowledge. A summary of recent methods is shown in **Table 1**.

### Recent ML Unsupervised Methods

A common task for unsupervised methods is to use the intrinsic structure of the data to find clusters of cells. The advantage of these approaches is that cells can be grouped in an automatic and unbiased manner and thus, have the potential to discover unknown cell populations.

The popular single-cell processing packages Seurat (42) and Scanpy (43) use a graph-based clustering approach combined with modularity optimization to group transcriptionally-similar cells together. Markers differentially expressed in each cluster can be found using different methods, including logistic regression. The cell identity of each cluster is assigned manually according to previous knowledge of cell-type specific markers. The main disadvantage of this approach is that the number of clusters depends on a resolution parameter assigned by the user (higher values will lead to a greater number of clusters) and thus, they may not faithfully reflect cell types.

The recently developed Single-Cell Clustering Assessment Framework (SCCAF) (24) generates an optimal number of clusters automatically. After the data has been clustered, SCCAF builds an ML classifier (logistic regression) using part of the data (training). By applying this model to the rest of the dataset (test), it iteratively merges clusters that appear indistinguishable to the ML classifier to produce the final optimum clustering. The output of the model is a weighted list of feature genes characteristic of every cluster that often include known markers for a given cell type and could potentially be used to detect common biomarkers of leukemic cell subsets from AML patients.

Another unsupervised method, single-cell consensus clustering (SC3) uses the first 4-7% * N (number of cells) **eigenvectors** to build multiple **k-means clustering** solutions (21). After hierarchical grouping, the final clustering is driven by the combination of multiple clustering solutions. The output is a list of marker genes that define each consensus cluster. While SC3 may not be the most sensitive method to find rare populations (such as LSCs), SC3 was successful in identifying clusters of prevalent genetic subclones with different mutations in myeloproliferative neoplasms (21). A disadvantage of this method is that it does not scale well for datasets with more than 5,000 cells (44).

A recent unsupervised method, weighted-nearest neighbour (WNN), was used to cluster cells using multiple data modalities (e.g. surface proteins and transcriptomes) measured in the same cell (25). This method uses **k-nearest neighbours** (kNN) to learn cell-specific modality “weights”. When applied to a multiomics dataset generated from human bone marrow samples (45), it showed that the combination of surface proteins and gene expression was superior for identifying cell populations than using one data modality alone. Multiomic single-cell technologies quantifying both surface proteins and transcriptomes of individual cells (e.g. CITE-seq), could be ideally applied to the identification of surface targets for the design of cell based immunotherapies (46).

Other unsupervised methods rely on Non-negative matrix factorization (NMF) methods (22, 23). These methods allow for the identification of cell types and, simultaneously, **cell states.** Given the great transcriptional heterogeneity seen in AML even within clonal populations carrying the same mutational patterns (16), it may be helpful to consider cell identities and activities separately when clustering leukemic populations. Moreover, NMF is potentially useful to identify LSC populations in AML, where the classical surface proteins defining primitive cell types are present in highly similar patterns to healthy HSCs, but a ‘malignant stem-like’ profile can still be identified (47).

### Recent ML Supervised Methods

Supervised methods to classify cell types exploit previously identified cell types and use either known marker genes or annotated reference datasets as an input to probabilistically assign new cells to a given category.

Some methods take a list of markers for each cell type as input (48). For example, CellAssign (26) uses predefined cell types input as a marker gene list to build a hierarchical model that produces a statistical classification of cells. This approach was used to delineate the composition of the tumour microenvironment in serial samples (treatment and relapse) from follicular lymphoma. Garnett (27) also takes as input a list of markers. The format of the input list permits accounting for cellular hierarchy (i.e, cell subtypes) and can include positive and negative markers to define cell types (27).

Other supervised methods use an annotated reference dataset to classify cell types but differ in the features and the ML methods used to train models (see **Table 1**). For instance, SingleCellNet (32) uses the most discriminative **gene pairs** (top pair transformation) to build a **random forest** classifier while methods such as scPred (36) and Moana (35) use principal components as features to fit a **support vector machine** (SVM). Some methods rely on one or several similarity metrics (such as SingleR (31)) and **k-nearest neighbours** (kNN) to map query datasets into a known reference [e.g. scmap (28) and scClassify (30)]. Other methods use the training dataset to build an **Artificial Neural Network** (ANN) model such as SuperCT (33) and ACTINN (34) with an input layer containing as many nodes as the number of genes in the training set and an output layer with nodes equal to the number of cell types. Interestingly, both ANN methods provide pre-trained models that could be used to classify new AML datasets.

An advantage of supervised ML approaches is that cell types are assigned probabilistically and some approaches allow for the possibility of an “unassigned” category (26–28, 32, 34). The unassigned label for cells that are absent or are very different in the reference dataset is key to limit misclassification and to allow the discovery of new cell types.

Algorithms such as CHETAH (29) and scClassify (30) allow for intermediate categories that can highlight populations with a mixture of identities as previously reported in AML (49). These methods are based on hierarchal correlation trees to classify test datasets (29, 30).

As more annotated single-cell datasets become available, the primary advantage of supervised methods is leveraging previous knowledge. Reference datasets of human bone marrow cells from healthy individuals are available from resources such as the Human Cell Atlas (50). Distinct cell populations or patient-specific tumour clones could be identified as unknown (because they are very different or absent in the reference data sets). As AML single-cell datasets become more abundant, they can be integrated with healthy single or multimodal references using ML methods (25).

A disadvantage of supervised methods is that they rely on known markers or accurate cell type annotations to build classification models. Often, markers for rare cell populations, such as LSCs, are unknown, not robust (51) or can be expressed by more than one cell type (15). Further, in many cases, annotation of single-cell datasets requires additional standardisation (29).

## Discussion

ML techniques are able to find non-trivial patterns in high-dimensional data (52). In fact, ML has already proven useful in identifying markers in bulk studies in prospectively isolated leukemic sub-populations (53, 54). However, ML has not reached its full potential for the characterisation of AML cell populations at single-cell resolution, partly due to the recent development of large datasets (5, 15–18).

Here we have reviewed tools to aid biomarker discovery using ML at single-cell level resolution. Many ML models explicitly quantify the contribution of individual features (genes) for a given classification. Importantly, genes identified in microarray data as important for classifying samples into “AML” or “no-AML” were not always differentially expressed (55). This means that traditional differential expression analysis could fail to identify biomarkers that are good predictors for assigning a class to a given group of cells (36). Thus, ML algorithms can find biomarkers that otherwise will be missed, expediting the design of suitable target combinations for immunotherapy.

Recently, it was shown that single-cell transcriptomics is capable of dissecting genetic subclones in AML, such as GATA2^R361C^, which cluster separately from normal hematopoietic cell types (16). This observation suggests that subclonal diversity in AML could be associated with distinct gene expression profiles which ML techniques can leverage to identify mutated populations. Some AML mutations create subtle differences in expression profiles (15–17) and isolating these populations represents an analytical challenge contemporary ML methods could address.

Moreover, recent experimental innovations allowing for the simultaneous quantitative assessment of cellular and molecular information at single-cell resolution promise to better dissect cell heterogeneity in AML. Particularly important is the ability to detect mutations in single cells combined with their transcriptional profiling, offering an unprecedent opportunity to identify specific leukemic cell populations (13, 15–17, 56, 57). For instance, the combination of single-cell transcriptomics and mutational profiles allowed the distinction of pre-leukemic clones, LSC and healthy HSC (17). ML such as SVM could be used next to identify molecules that maximise this classification as done before for bulk RNA-seq and microarray data (53).

In addition, the identification of mutant and non-mutant cells allows for applying ML methods to both all and only mutated cells to further characterise subpopulations (16), and can be used to fine-tune ML classification algorithms. For instance, a two-step ML classification strategy was applied to bone marrow samples of AML patients (15). First, a fraction of mutant cells was identified by genotyping and these were classified into one of six normal haematopoietic cell types (monocyte-like, progenitor-like, etc.). Subsequently, these malignant cell types were incorporated as additional classes in a second classifier that successfully identified mutant and normal cells from their transcriptome profiles.

The simultaneous characterization of surface proteins at single-cell resolution (46) is especially important for isolation of heterogeneous cell populations. There are some analytical challenges with the integration of multiple data modalities (58), but combining different data types from the same cell has already shown to improve the identification of cell populations in AML datasets (16, 18) and healthy bone marrow samples (25), thus we anticipate that multimodal datasets will improve the performance of ML models in isolating specific cell populations and may facilitate the identification of relevant surface targets for precision immunotherapy.

All the methods reviewed here will incur a certain degree of **underfitting** and **overfitting**. Thus, it is wise to compare algorithms in the initial cell composition assessment. Some, such as hierarchical methods, are potentially more suitable for AML samples, where there is an intrinsic hierarchy shared with normal hematopoietic development (**Figure 1B**). Also, methods that enable the recognition of intermediate cell types, mixed identities or different cell states would be more suitable for the identification of abnormally differentiated leukemic cells, known to be characteristic of AML (49).

Finally, we anticipate that single-cell resolution phenotyping will be important for the design of cell-based immunotherapy combinatorial strategies accounting for clonality and differentiation states of AML populations, with ML likely playing a pivotal role in the selection of optimal therapeutic targets for the design of personalised workflows tailored to each patient.

## Author Contributions

All authors contributed to the article and approved the submitted version. YC conducted literature review and wrote the manuscript in consultation with RP. SG and AG critically revised the work.

## Funding

This work was supported by the NIHR GOSH BRC. Part of this work was funded by the NIHR HS&DR Programme (14/21/45) and supported by the NIHR GOSH BRC. AG is supported by the Leukaemia UK John Goldman Fellowship, (2018/JGF/003), the Rosetrees Trust fund (M700), the Academy of Medical Sciences Springboard Award (SBF004\1025) and the Cancer Research UK (C65772/A29812).

## Disclaimer

The views expressed are those of the authors and not necessarily those of the NHS, the NIHR or the Department of Health.

## Conflict of Interest

The authors declare that the research was conducted in the absence of any commercial or financial relationships that could be construed as a potential conflict of interest.

## Glossary

**Artificial Neural Network (ANN):** A type of supervised learning model where multiple simple functions (artificial neurons) are connected in layers, which sequentially process information. ANNs contain an input layer which passes the information to several “hidden” layers, these are activated depending on the input and feed this information to the output layer, which reflects the assigned class. Deep ANNs are those with many hidden layers.

**Annotated Reference Dataset:** A (single cell) expression dataset, where the cell types of all cells are known, e.g. through experimental validation. Reference datasets are useful to assign likely labels (classify) to new cells (query) that are similar to cells in the reference.

**Cell state:** The cellular activities a cell is carrying out at a given moment. These can be general (e.g. hypoxia response) or specialised (e.g. cycling).

**Cell type:** The kind of cell, e.g. a Red Blood Cell. Cell types are commonly associated with specialised functions, markers and histology. However, it is important to note that cell types are often fluid or non-constant and distinguishing two similar cell types can be difficult.

**Classes and Clusters:** Both describe grouping data points by measurements made during experiments. The key difference is that clusters refer to groupings obtained through unsupervised learning, whereas classes refer to groups from supervised learning. Importantly, classification is able to assign class-names (based on the training dataset), whereas clusters are “nameless”.

**Eigenvector and Eigenvalue:** Eigenvectors are the vectors which do not change in direction if a matrix is linearly transformed; the eigenvalue is the scalar denoting by how much the eigenvector has changed in magnitude after transformation. In this way eigenvectors and eigenvalues can represent a matrix (eigen decomposition), encoding the fundamental structure of the matrix. An example use of eigen decomposition is Principal Component Analysis.

**Gene pairs (top pair transformation):** Transformation based on comparing the expression of pairs of genes within each cell, limited to genes that are preferentially expressed in each cell type defined in the training data, as well as those genes that are specifically under-expressed in each type.

**K-nearest neighbour algorithm:** Training datapoints of known classes are mapped into a (usually dimensionally reduced) space. New datapoints are then mapped into the same space and a class is assigned to each as the most frequent class of their k (e.g. 7) nearest neighbours.

**K-means clustering algorithm:** Method that aims to partition n observations into k clusters such that each observation belongs to the cluster with the nearest mean.

**Marker:** A characteristic protein, often expressed on the surface of a cell, or gene, e.g. a transcription factor, that can be used to mark a specific cell type experimentally.

**Overfitting:** Occurs when a model fits a particular dataset too closely, it will then fail to generalise to unseen data.

**Random Forests and Decision Trees:** Decision Trees learn a “yes-no flow chart” to sequentially partition data until a classification is reached; individual decision trees are prone to overfitting. Random Forests are multiple independent decision trees trained together. Classification output is the average output of all trees, overcoming overfitting seen in an individual tree.

**Single cell gene expression matrix: **The processed data obtained from single cell expression experiments is usually represented by a gene expression matrix. This is a large table where every row represents a gene, and every column the reads measured in a single cell.

**Supervised Learning:** A collection of machine learning approaches where characteristic labels (classifications) are learned from data annotated with known classes.

**Support Vector Machine (SVM):** Is a supervised learning algorithm that aims to find the hyperplane that best separates two classes, i.e. goes ”right through the middle”. SVM can be extended to non-linearly-separable data using a kernel function that maps the data to a higher dimensional space in which it is linearly separable.

**Training Data and Test Data: **When training a machine learning algorithm datasets should be split into training and test data. The model is learned using the training data. Test data are a subsection of the original dataset, that the model has not encountered in training, and can be used to approximate the model’s expected performance on unseen data.

**Underfitting:** Occurs when a model does not adequately learn the underlying structure of the data.

**Unsupervised Learning:** A collection of machine learning approaches that learn a pattern in the unlabelled data.
